# Genome Characterization of Two Novel Lactococcus lactis Phages vL_296 and vL_20A

**DOI:** 10.32607/actanaturae.27468

**Published:** 2024

**Authors:** T. A. Chuksina, A. A. Fatkulin, N. P. Sorokina, I. T. Smykov, E. V. Kuraeva, E. S. Masagnaya, K. A. Smagina, M. Yu. Shkurnikov

**Affiliations:** Department of Biology and Biotechnology, HSE University, Moscow, 101000 Russian Federation; V.M. Gorbatov Federal Research Center for Food Systems, Moscow, 109316 Russian Federation

**Keywords:** bacteriophage, CRISPR-Cas, cheese making, starter cultures, One Health

## Abstract

Fermented dairy products are produced using starter cultures. They ferment milk to create products with a certain texture, aroma, and taste. However, the lactic acid bacteria used in this production are prone to bacteriophage infection. We examined the genomes of two newly discovered bacteriophage species that were isolated from cheese whey during the cheesemaking process. We have determined the species and the lytic spectrum of these bacteriophages. Phages vL_20A and vL_296 were isolated using lactococcal indicator cultures. They have unique lytic spectra: of the 21 possible identified host bacteria, only four are shared amongst them. The vL_20A and vL_296 genomes comprise linear double-stranded DNA lengths with 21,909 and 22,667 nucleotide pairs, respectively. *Lactococcus phage bIL67 *(ANI 93.3 and 92.6, respectively) is the closest to the phages vL_20A and vL_296. The analysis of the CRISPR spacers in the genomes of starter cultures did not reveal any phage-specific vL_20A or vL_296 among them. This study highlights the biodiversity of* L. lactis *phages, their widespread presence in dairy products, and their virulence. However, the virulence of phages is balanced by the presence of a significant number of bacterial strains with different sensitivities to phages in the starter cultures due to the bacterial immune system.

## INTRODUCTION

The production of fermented dairy products such as cheeses and yogurts is based on the use of starter cultures. They ferment milk, creating a product with a certain texture, aroma, and taste [1]. However, the lactic acid bacteria used in production are susceptible to bacteriophage infection [2]. Dairy factories represent a specific, isolated ecological niche for the bacteriophages of lactic acid bacteria, because lactobacilli are naturally present in raw milk and artificially introduced into pasteurized milk via bacterial starter cultures [3].

High levels of lactic acid fermentation prevent the development of extraneous and pathogenic microorganisms in milk, which are inactivated during pasteurization or trapped in milk after pasteurization, and also determine the population characteristics of the microbiome of dairy products by increasing acidity and specific antagonism against non-lactic acid bacteria. Phage infection can adversely affect the fermentation and growth of bacterial cultures [1]. If phages attack the starter culture, the fermentation process may slow down or even stop altogether. As a result, there is a risk of developing pathogenic microflora and the emergence of deviations in taste, aroma, and texture [4].

The composition of starter cultures for many fermented dairy products and cheeses includes* Lactococci *(*Lactococcus lactis *sps.*, L. cremoris*). Therefore, bacteriophages lysing *Lactococci *are widely used in the dairy industry. Cheesemaking is the most vulnerable from the point of view of phage attacks. This is due to the fact that the mildest milk pasteurization mode is used in cheese production (temperature 72–76°C with exposure of 20–5 s), and part of the bacteriophage population of raw milk is not destroyed. In addition, the serum formed during production almost always contains significant amounts of virions and is a significant source of bacteriophage spread in dairy factories, which are found in various objects, including production leaven, equipment, sanitary clothing, and exposed body parts of workers [5]. After the report on lactococcal bacteriophages in the 1930s [6] and the following numerous studies of this phenomenon, phagolysis is now considered a constant nuisance that is difficult to eliminate in the dairy industry.

Phage-resistant strains of lactic acid bacteria are selected to protect lactic acid bacteria from bacteriophages, and batches of starter cultures are systematically rotated [7], which points to the pressing need to study the phage resistance and the phagotype of lactococcal collection cultures. The effectiveness in selecting phage-resistant lactococcal cultures largely depends on the composition of the set of phages used and the spectrum of their lytic action. This indicates that systematic research into the phage background of dairy factories is needed. The phage–host interactions in cheese production are also a subject of interest from the perspective of the One Health paradigm, which implies a comprehensive, unified approach aimed at sustainable balance and optimization of human, animal, and their shared environmental health, including the ecosystem of dairy factories.

Bacteriophages are the most common viruses found on Earth. Most free-living bacteria are infected by phages. This is evidenced by the presence of prophages in most bacterial genomes [8, 9]. Bacteria have developed many mechanisms for anti-bacteriophage defense, which can be referred to as the “prokaryotic immune system” [10]. These mechanisms can be further separated into the innate and adaptive “prokaryotic immune systems” [11]. Classic examples of innate immunity include the restriction modification (R-M) [12] and phage infection abortion (Abi) systems [13]. However, many additional innate immune mechanisms have recently been discovered, highlighting the strong selective pressure exerted by phages on microbial communities [14,15].

The CRISPR-Cas system is the only “adaptive” prokaryotic immune system there is. It allows bacteria to incorporate short DNA sequences from phages into a special CRISPR cassette. Upon meeting a phage, transcribed spacers bind to the DNA of the phage and direct its degradation using Cas proteins [16].

In this study, we examined the genomes of two new bacteriophage species isolated during cheese production. We determined the species and the lytic spectra of the phages and analyzed their possible virulence mechanisms and sensitivity to the CRISPR-Cas system in the main starter cultures.

## EXPERIMENTAL

**Phage isolation and purification**

Bacterial strains from the Collection of lactic acid bacteria for cheesemaking and phages were used in this study (All-Russia Research Institute of the Cheese and Butter Industries, V.M. Gorbatov Research Center for Food Systems, Russian Academy of Sciences).

Bacteriophages were isolated from cheese whey samples. The bacteriophage vL_20A was isolated from whey obtained during the manufacturing of semi-hard cheese at the Pereslavl Cheese Factory (Yaroslavl Region, Russia) on January 6, 1985, and spread on a sensitive culture of *L. lactis *subsp. *lactis* 393-8. The bacteriophage vL_296 was isolated from whey obtained during the manufacturing of semihard cheese at the Yugovsky Dairy Products Plant (Perm Krai, Russia) on July 6, 2022, and spread on a sensitive culture of *L. lactis *subsp. *lactis *345-8.

The sensitive culture was grown on a M17 medium containing lactose (HiMedia, India). Serum samples were filtered through a sterile filter with a pore size of 0.45 μm.

The surface seeding method was used to isolate the bacteriophages: 0.1 mL of the *L. lactis *subsp.* lactis *culture was applied on Petri dishes containing the dried solid medium M17 (1.5% agar) in the phase of logarithmic growth, rubbed in with a glass spatula, and left for 10–15 min to absorb moisture into the agar. A drop of filtered serum was then applied on the Petri dish, covered with a lid, and left to rest for 10–15 min at room temperature. Next, the Petri dishes were flipped over and thermostated for 16–18 h at a temperature of 30 ± 1°C. In the presence of lysis zones at the site where serum was applied, a piece of agar from the lysis zone was placed in a test tube containing 3 mL of the M17 medium, thoroughly shaken, and kept for 24 h at 4 ± 2°C to ensure a more complete release of phage particles from the agar. Next, a drop of the medium from the test tube was applied on a fresh lawn of the culture and thermostated for 16–18 h. To obtain a pure bacteriophage, isolated mixtures of bacteriophages were titrated using a two-layer agar method: 0.1 mL of the culture and 0.1 mL of 10-fold dilutions of the phage mixture were introduced into test tubes containing 3 cm^3^ of semi-liquid M17 agar (0.6% agar); the suspension was poured into a dish containing a dense medium and incubated for 18–24 h at 30 ± 1°C. Pieces of agar from individual negative colonies (plaques) were used to accumulate phages in a liquid medium containing a sensitive culture. The cultured bacteriophages were filtered through a sterile filter with a pore size of 0.22 μm and stored at 4 ± 2°C.

**Phage host range**

The lytic activity of the phages against 35 strains of* L. lactis *subsp. *lactis*, 35 strains of *L. cremoris*, and 35 strains of *L. lactis *subsp. *lactis *biovar. diacetylactis was determined by cultivation on a double-layer agar in culture plates [17]. The sensitivity of lactococci to phages was determined by the presence of a plaque at that spot. The results were divided into two categories: with plaque (+) and without plaque (−).

**Transmission electron microscopy**

Phage samples were fixed in a 1.5% glutaraldehyde solution in 0.1 M Sorenson phosphate buffer (pH 7.2) for 20 min at room temperature. Subsequently, 5 μL of the fixed sample was transferred to a supporting copper mesh (mesh-400) coated with a nitrocellulose (parlodium) film and kept for 2 min to deposit dispersed particles on the film surface. The contrast in a sample was increased by negative contrast [18, 19]. To achieve this, a drop (2 μL) of a 2% uranyl acetate solution was pipetted onto a drop of a fixed sample located on a grid and held for 4 min. After exposure, the excess solution was removed from the mesh surface using filter paper and placed in a vacuum chamber for final drying at room temperature.

Electron microscopic studies of bacteriophage morphology were conducted using an EM-410 transmission electron microscope (Philips, Netherlands) operated at 40 kV. The images were captured on a Fujicolor C-200 film (FUJIFILM Corporation, Japan).

**Phage DNA isolation and sequencing**

A precipitate of the solution (4% PEG-6000, 1 M NaCl) was added to the bacterial lysate samples. Incubation was conducted at 4°C for 3 h. After the incubation, the tubes were centrifuged (12,000 *g*) for 15 min at 4°C. The supernatant was selected, and the precipitate was resuspended in 180 μL of PBS. Then, 1.25 μL of Proteinase K (20 mg/mL) was added to the samples and the mixture was incubated at 56°C for 1.5 h without shaking. DNA was isolated using a QIAamp Viral DNA kit (Qiagen, Germany), according to the manufacturer’s protocol. The DNA concentrations and quality were evaluated using Nanodrop and Qubit.

The NEBNext Ultra II DNA Library Prep Kit for Illumina (New England BioLabs, USA) was used to create DNA libraries according to the manufacturer’s protocol. Sequencing of the obtained libraries was performed on a NovaSeq 6000 high-performance sequencer (Illumina, USA).

**Genome analysis**

The FastQC 0.12.1 software was used to assess the quality of raw reads. They were then preprocessed using the fastp 0.23.2 tool. Additionally, taxonomic read classification was performed with the standard Kraken 2 database. Reads were assembled using SPAdes 4.0.0. Host genomes were obtained by applying the – isolate flag, while the – metaviral flag was specified for viral genome assembly. The QUAST 5.2.0 tool was used to evaluate the genome assembly quality.

Viral genomes were validated using CheckV 1.0.1, and complete ones were subsequently annotated with Pharokka 1.6.1.

To analyze CRISPR spacers, 562 genomes from the starter cultures of the species *Lacticaseibacillus casei*,* Lacticaseibacillus paracasei*, *Lacticaseibacillus rhamnosus*,* Lactiplantibacillus plantarum*, *Lactobacillus helveticus*, and *Propionibacterium freudenreichii *from the NCBI GenBank database were used [20].

Bacterial genomes were examined for the presence of immune systems using the MinCED 0.4.2 and PADLOC 2.0.0 software tools.

**Data accession**

The whole-genome sequences of vL-20A and vL_296 were deposited into GenBank under the accession numbers PQ062249 and PQ062250.

## RESULTS

**Isolation and the morphological characteristics of phages**

**Fig. 1:**
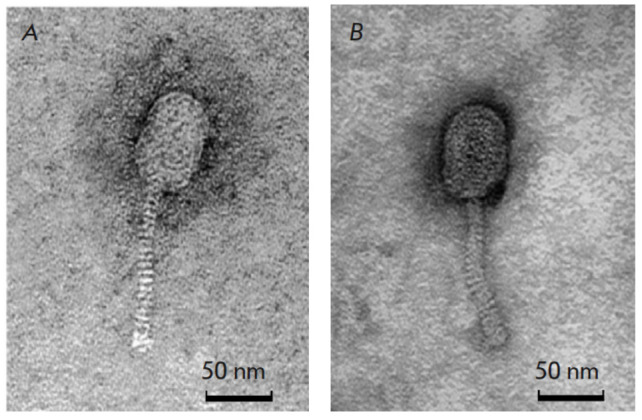
Transmission electron microscopy images of* Lactococcus lactis *phage vL_20A (*A*) and *Lactococcus lactis *phage vL_296 (*B*)

The phages vL_20A and vL_296 were isolated from subcutaneous serum using the indicator cultures* L. lactis *subsp. *lactis *393-8 and *L. lactis *subsp. *lactis* 345-8 as host bacteria. Transmission electron microscopy (*Fig. 1*) revealed that phage vL_20A had an icosahedral head with a diameter of 39 ± 3 nm and a tail 107 ± 6 nm long. In turn, phage vL_296 had an icosahedral head with a diameter of 45 ± 4 nm and a tail 125 ± 8 nm long. This morphology is similar to that of *Caudoviricetes*. According to the International Code of Virus Classification and Nomenclature [21], these phages were designated as *L. lactis *phage vL_20A and *L. lactis *phage vL_296 (Viruses; Duplodnaviria; Heunggongvirae; Uroviricota; Caudoviricetes; Ceduovirus; Ceduovirus vL_20A and vL_296).

**Host range test analysis**

**Fig. 2:**
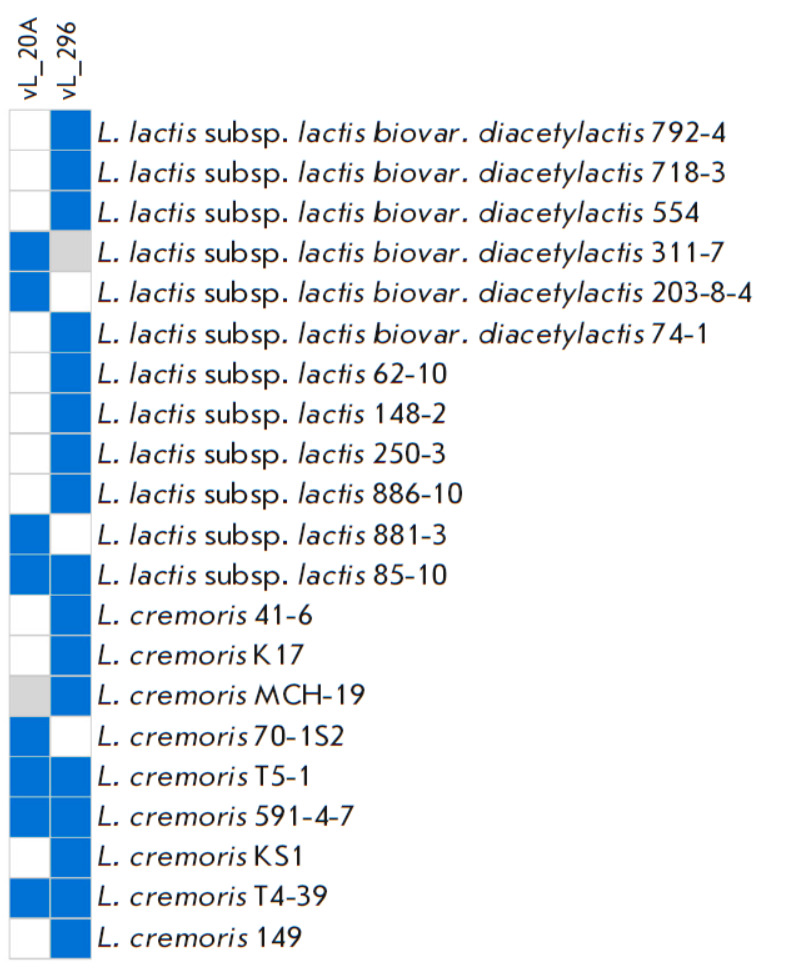
Host ranges of the vL_20A and vL_296 phages. Blue: presence of plaque; white: absence of plaque; gray: no analysis was conducted

Four strains of *L. cremoris *were lysed by vL_20A; eight, by vL_296 (Fig. 2). The lytic activity against *L. cremoris* was 11.8% (4/34) for vL_20A and 22.9% (8/35) for vL_296. The lytic activities against *L. lactis *subsp.* lactis *were 5.7% (2/35) and 14.3% (5/35), respectively. For *L. lactis *subsp. *lactis *biovar. diacetylactis, the lytic activities of vL_20A and vL_296 were 5.7% (2/35) and 14.3% (4/28), respectively. It can be noted that vL_20A and vL_296 have unique host ranges. Of the 21 identified host bacteria, only four were common between them (*L. cremoris *T4-39, *L. cremoris *591-4-7, *L. cremoris* T5-1, and *L. lactis *subsp. *lactis *85-10).

**Genome analysis**

**Fig. 3:**
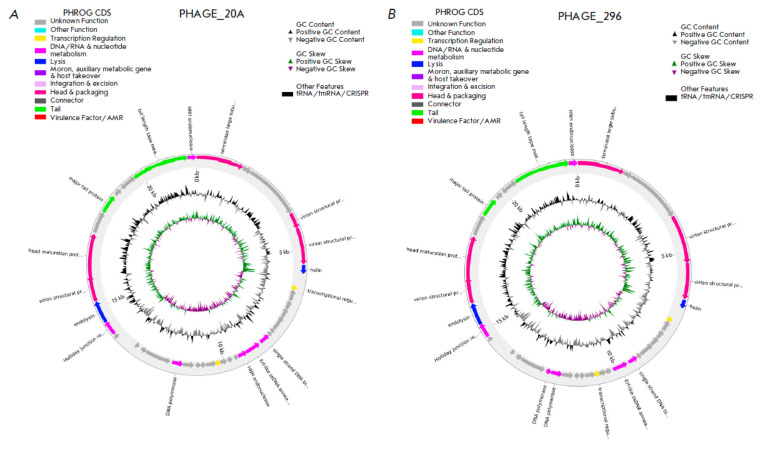
Visualization of the known functional proteins encoded in the genome of *Lactococcus lactis *phage vL_20A (*A*) and *Lactococcus lactis *phage vL_296 (*B*)

The whole-genome sequences of the vL_20A and vL_296 genomes were obtained using the Illumina NovaSeq 6000 platform. Their genomes consisted of linear double-stranded DNA with a length of 21,909 bp (GC 35.75%) and 22,667 bp (GC 35.89%), respectively. Forty-seven open reading frames (ORFs) were predicted for vL_20A and 43 ORFs, for vL_296; of those, 11 ORFs were similar to the genes encoding known functional proteins (*Fig. 3*), while the remaining ORFs encoded putative proteins.

According to functional activity, all the predicted proteins were divided into four groups (*Fig. 3*): DNA metabolism proteins (2 ORF each), packing and head formation proteins (5 ORF each), lysis proteins (two ORFs each), and tail proteins (two ORFs each). The remaining ORFs presumably encoded proteins with unknown activity. Searching across the VFDB and CARD databases revealed no virulence or antibiotic resistance genes.

**Comparative genome analysis**

The main criterion for identifying new virus species was the identity of the phage genome with other species within the genus by less than 95% [21]. To determine the genomic similarity of vL_20A and vL_296 to other phages, we first performed a BLASTn search across the NCBI database. The genomes of the *L. phage *genus of viruses were obtained from the Nucleotide NCBI database, and the average nucleotide identity (ANI) was evaluated.

**Fig. 4:**
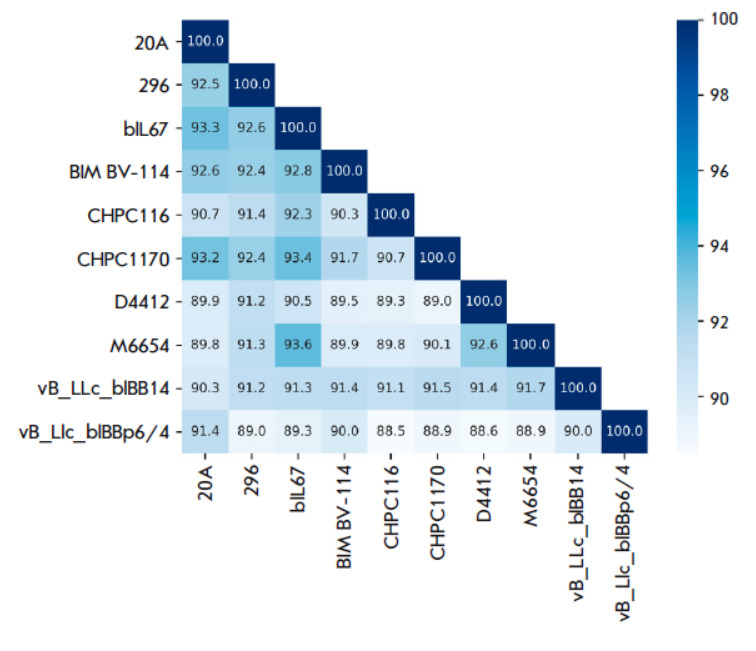
Heatmap of average nucleotide identity between ten genomes of *Lactococcus lactis *phages

**Fig. 5:**
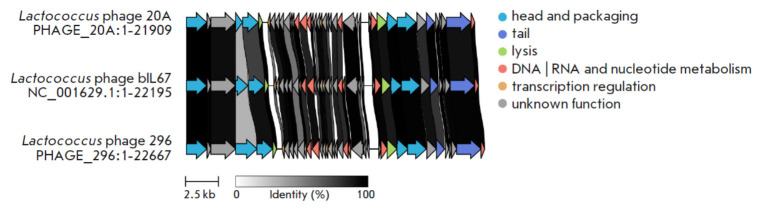
Schematic diagram of the genome structure of the *Lactococcus lactis phage vL_20A *and *Lactococcus lactis phage vL_296 *compared to the most similar *Lactococcus phage bIL67*

*Figure 4* shows the ANI for the ten most similar phages among the 254 analyzed genomes. The vL_20A and vL_296 phages were the closest to the* L. phage *bIL67 (ANI 93.3 and 92.6, respectively). Note that the ANI between vL_20A and vL_296 is lower than that of the *L. phage *bIL67 and is 92.5 (*Fig. 5*). It can be assumed that vL_20A and vL_296 represent separate species not described previously.

**Fig. 6:**
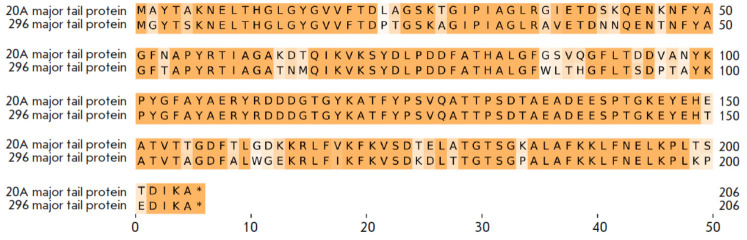
Alignment of major tail protein DNA sequences in the vL_20A and vL_296 genomes

The ANI between these two phages is only 92.5. When we compare the vL_20A and vL_296 genomes, we find a high number of polymorphisms in the major tail protein, which is crucial for the phage to attach to the host cell (*Fig. 6*). Furthermore, variations in the nucleotide sequence of phages may potentially impact the efficiency of bacterial cell defense mechanisms designed to break down the viral genome.

**CRISPR spacer analysis**

**Fig. 7:**
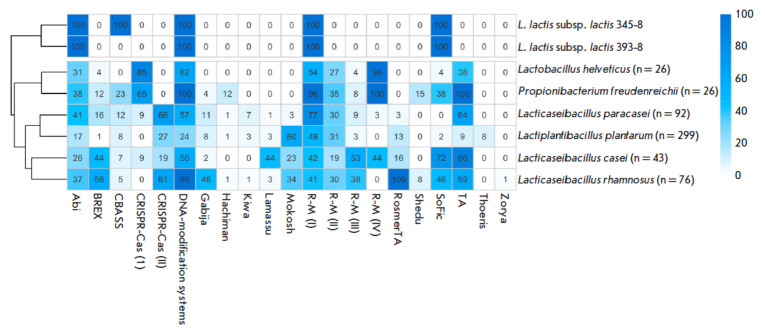
The immune system of lactic acid bacteria. The cells indicate the frequency of the occurrence (%) of the immunity mechanism in starter cultures. The genus name indicates the number of analyzed genomes

Bacteria possess a range of defense mechanisms, including the widespread defense system against phages, CRISPR-Cas. CRISPR spacers are involved in adaptive immunity, ensuring complementary binding of RNA to the nucleic acids of foreign elements and their subsequent destruction by Cas proteins. This system is present in most starter cultures (*Fig. 7*).

We analyzed the presence of spacers specific to the vL_20A and vL_296 phages in the genomes of starter cultures. Among the 562 genomes analyzed, it was impossible to identify any spacers specific to the vL_20A and vL_296 phages. It can be the case that the new phage species we have identified had not been in contact with the analyzed starter cultures for a long time.

## DISCUSSION

Phage attacks on the acid-forming microflora of cheeses are extremely dangerous from a product safety perspective, since there is a threat of more intensive development of residual post-pasteurization microflora. To reduce the risk of a release of substandard and dangerous products to consumer health due to phage attacks, it is particularly important to limit the reproduction of phages through the use of multi-strain starter cultures, as well as their systematic rotation and the inclusion of phage-resistant cultures in the starter microflora.

In order to be able to select phage-resistant strains in starter cultures, the diversity and properties of phages capable of infecting starter cultures need to be studied. In particular, the phages described in this paper were isolated from cheese whey with an interval of 37 years and caused problems with the fermentation of raw materials. This indicates that phages and bacterial strains that are sensitive to them continue to exist in starter cultures, as shown in previous studies [22]. Considering that the ANI similarity level for the newly isolated phages was significantly below 95, it is fair to assume that we have described two new phage species for the first time.

The closest representative of the genus for these phages is the species *L. phage *biL67 [23]. We established that the genomes of the new phages* Lactococcus lactis *phage vL_20A and *L. lactis *phage vL_296 are linear double-stranded portions of DNA with lengths of 21,909 and 22,667 bp, respectively. In the genomes, 47 and 43 ORFs can be distinguished for vL_20A and vL_296, of which 11 are similar to the genes encoding known functional proteins.

The lytic spectra of the phages are quite narrow and virtually do not overlap. Only four common strains of the host bacteria could be distinguished:* L. cremoris *T4-39, *L. cremoris *591-4-7, *L. cremoris* T5-1, and *L. lactis *subsp. *lactis *85-10. These data differ from the results obtained by Stuer-Lauridsen et al., where most of the studied *L. lactis *phages were able to lyse 10–90% of the strains [24]. This can be attributed to the differences in the sources of the dairy products from which the phages were isolated in these two studies.

The analysis of CRISPR cassettes of starter bacteria failed to identify any spacer specific to the vL_20A and vL_296 phages. It can be assumed that the new phage species we have identified had not been in contact with the analyzed starter cultures for a long time. Nevertheless, CRISPR/Cas systems of types 1 and 2 have been found in the genomes of many of them. This suggests that immunity against these phages may develop when they are encountered.

## CONCLUSIONS

Today, we need to move towards sustainable, inclusive, and independent agri-food systems. This can be achieved by regarding the food system as a continuous and interconnected chain, where risks are monitored and assessed at every stage, from raw materials to finished products. In this chain, the phage health of the production environment is the main cause of disrupted sustainability in the production of fermented dairy products.

Our study highlights the biodiversity of phages isolated from cheese whey and confirms their widespread presence in dairy factories and their virulence. However, the presence of bacterial strains with varying degrees of sensitivity to phages in the starter cultures balances this danger through bacterial immunity systems.

The high level of resistance of starter cultures to phage infection may prevent the mass reproduction of phages in multicomponent starter cultures. This explains why phages are found in fermented dairy products without acidification problems. However, the simultaneous presence of various active phages within a single starter culture can sometimes lead to a defective product. Additional research is needed to better understand the ecological role of phages and assess their impact on fermentation. The abundance of bacteriophages in dairy plants infecting starter cultures once again highlights the importance of developing strategies to combat phages in the dairy industry.

## Abbreviations

R-M: restriction-modification system
Abi: phage infection abortion system
